# Dissection of Genetic Basis Underpinning Kernel Weight-Related Traits in Common Wheat

**DOI:** 10.3390/plants10040713

**Published:** 2021-04-07

**Authors:** Shunda Li, Liang Wang, Yaning Meng, Yuanfeng Hao, Hongxin Xu, Min Hao, Suque Lan, Yingjun Zhang, Liangjie Lv, Kai Zhang, Xiaohui Peng, Caixia Lan, Xingpu Li, Yelun Zhang

**Affiliations:** 1 College of Plant Science & Technology, Huazhong Agricultural University, No. 1, Shizishan street, Hongshan district, Wuhan 430070, China; 15071308591@163.com (S.L.); wangzi651@163.com (L.W.); hmmail.hzau.edu.cn@webmail.hzau.edu.cn (M.H.); 2 Institute of Cash Crops, Hebei Academy of Agriculture and Forestry Sciences, Shijiazhuang 050051, China; yaningmeng@126.com; 3 Institute of Crop Science, National Wheat Improvement Center/The National Key Facility for Crop Gene Resources and Genetic Improvement, Chinese Academy of Agricultural Sciences (CAAS), Zhongguancun South Street 12, Beijing 100081, China; haoyuanfeng@caas.cn; 4 A State Key Laboratory of Crop Stress Adaptation and Improvement, State Key Laboratory of Cotton Biology, School of Life Sciences, Henan University, Kaifeng 475004, China; hongxingxu@henu.edu.cn; 5 The Key Laboratory of Crop Genetics and Breeding of Hebei Province, Institute of Cereal and Oil Crops, Hebei Academy of Agricultural and Forestry Sciences, Shijiazhuang 050035, China; lansq66@126.com (S.L.); zhangyingjun1977@163.com (Y.Z.); liangjie_lv@163.com (L.L.); kai_zhang_zk@outlook.com (K.Z.); wiwin.ambarwulan@big.go.id (X.P.)

**Keywords:** thousand kernel weight, grain length, grain width, QTL, common wheat

## Abstract

Genetic dissection kernel weight-related traits is of great significance for improving wheat yield potential. As one of the three major yield components of wheat, thousand kernel weight (TKW) was mainly affected by grain length (GL) and grain width (GW). To uncover the key loci for these traits, we carried out a quantitative trait loci (QTL) analysis of an F_6_ recombinant inbred lines (RILs) population derived from a cross of Henong 5290 (small grain) and 06Dn23 (big grain) with a 50 K single nucleotide polymorphism (SNP) array. A total of 17 stable and big effect QTL, including 5 for TKW, 8 for GL and 4 for GW, were detected on the chromosomes 1B, 2A, 2B, 2D, 4B, 5A, 6A and 6D, respectively. Among these, there were two co-located loci for three traits that were mapped on the chromosome 4BS and 6AL. The QTL on 6AL was the most stable locus and explained 15.4–24.8%, 4.1–8.8% and 15.7–24.4% of TKW, GW and GL variance, respectively. In addition, two more major QTL of GL were located on chromosome arm 2BL and 2DL, accounting for 9.7–17.8% and 13.6–19.8% of phenotypic variance, respectively. In this study, we found one novel co-located QTL associated with GL and TKW in 2DL, *QGl.haaf-2DL.2*/*QTkw.haaf-2DL.2*, which could explain 13.6–19.8% and 9.8–10.7% phenotypic variance, respectively. Genetic regions and linked markers of these stable QTL will help to further refine mapping of the corresponding loci and marker-assisted selection (MAS) breeding for wheat grain yield potential improvement.

## 1. Introduction

Wheat is one of the most important food crops, which has the largest cultivated area worldwide (http://faostat.fao.org, (accessed on 1 December 2020)). It is the staple food of more than 35% of the world’s population, which provides one fifth of the daily intake of calories and protein. [1]. In order to meet the higher demand of wheat due to the increasing human population, rapid urbanization and sharp climate change, it has been estimated that global production is necessary to grow at a rate of 1% per year in the future [2]. Therefore, wheat yield improvement is still the top priority in wheat breeding programs to guarantee global food security.

Wheat yield is a complex trait consisting of three main components—the number of spikes per area, kernel number per spikes, and thousand kernel weight (TKW). Among them, TKW has relatively high heritability [3] and is a quantitative trait. As one key component of grain yield, TKW is mainly influenced by grain size and grain filling [4]. Grain size provides the room for grain filling and can be broken into grain length (GL), grain width (GW), grain thickness (GT) and grain surface area. Compared with GL, the other three components are more sensitive to the environment as a result of their establishment in the later stage of grain development [5,6,7]. Grain filling directly affects dry matter accumulation in grains and can be split into grain filling rate and duration, which could be severely hindered under adversity stress [8,9,10,11,12,13]. Although these components are susceptible to environmental impact, their inheritance are relatively stable compared with the total yield of wheat. Therefore, TKW and its related traits are often used in genetic analysis of wheat grain yield.

In recent years, large advancement was made in wheat genomics and multiple high-quality wheat genomes called the wheat “pan genome” [14]. Ascribable to the release of the reference genome, high-throughput single nucleotide polymorphism (SNP) genotyping is progressively applied to wheat genetic analysis, including SNP chips and simplified genome sequencing [15,16,17,18,19]. Rapid and high-efficiency genotyping greatly accelerate the discovery of genetic loci for wheat. Up to now, a number of loci associated with grain weight have been identified on 21 chromosomes of wheat based on the linkage mapping approach and genome-wide associated study (GWAS) approach [5,15,20,21,22,23,24,25,26,27,28,29,30,31,32,33,34,35,36,37,38,39]. Some major quantitative trait loci (QTLs) were developed to do fine mapping in a previous study. [4,35,40,41,42,43]. Nevertheless, there is not yet a grain weight-related gene isolated via map-based cloning in wheat, due to its large and complex genome and as well as minor effect [5,44]. With the advancement of more high-quality reference genomes of common wheat and its relatives [45,46,47,48,49], the efficiency of map-based cloning will become faster. Therefore, it is of necessity to rapidly excavate and validate more stable and major QTLs for grain weight.

In this study, we selected HeNong 5290 (hereafter HN5290) with small grain and 06Dn23 with big grain to make an F_6_ recombinant inbred lines (RILs) population for understanding the genetic basis of controlling TKW, GL and GW. The purposes were to detect the stable and co-located loci for TKW, GL and GW and analyze the relationships between detected co-located loci on the three traits.

## 2. Results

### 2.1. Phenotypic Statistics

**Figure 1:**
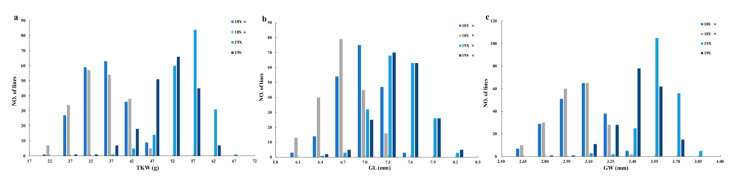
(**a**) Frequency distributions for thousand kernel weight (TKW), (**b**) grain length (GL) and (**c**) grain width (GW), respectively, of 194 F_6_ recombinant inbred lines (RILs) derived from the cross of HN5290×06Dn23 and their parents over four environments (18S, 18Y, 19X and 19S). The 18S and 19S, Shijiazhuang, China 2017–2018 and 2018–2019; 18Y, GaoYi, China 2017–2018; 19X, XinXiang, China 2018–2019; * *p* > 0.05 based on Shapiro–Wilk test.

Phenotypic data were obtained from the field trials at three different locations over two continuous years (as such, four environments). The mean value of TKW, GL and GW for 06Dn23 ranged from 46.3–70.8 g, 7.52–8.33 and 3.37–3.89 mm, respectively, while it was 25.9–45.8 g, 6.08–6.67 and 2.79–3.39 mm for HN5290, respectively (Table 1). The TKW, GL and GW of the RILs ranged from 17.6–67.0 g, 6.85–7.60 and 3.09–3.65 mm, respectively (Table 1). Based on a *t*-test, 06Dn23 showed significantly higher values for three traits over all environments than HN5290 (Table 1). The coefficient of variation of TKW ranged from 7.7–15.8%. In addition, the highly significant difference interactions between genotypes and environments for grain weight related-traits among RILs were detected by ANOVA analysis (Table 2). The frequency distribution of phenotypic values of TKW, GL and GW showed a continuous and normal distribution (Figure 1) with an exception of the 2018 crop cycle, while there was a significant distorted distribution of the three grain-related traits in the 2018 crop cycle, indicating that this might be due to the high temperature during the grain filling period in 2018 (Supplementary Table S3).

In addition, the correlation coefficients of grain-related traits were extremely significant under four environments, while it was 0.78–0.87 for GL among different environments and it was also significantly correlated with TKW and GW, which were 0.42–0.71 and 0.21–0.57, respectively. Interestingly, TKW was significantly positively correlated with GW (*r* = 0.54–0.94), while the correlation between GL and GW was weak (*r* = 0.21–0.57) (Table 3). The broad-sense heritability of TKW, GL and GW was 0.80, 0.89 and 0.75, respectively (Table 2), indicating that GL was more insensitive to the environmental variation than TKW and GW in the present study.

### 2.2. Linkage Map Construction

A total of 18,357 SNP loci were polymorphic between two parents. Among them, 4655 SNPs with a high missing rate (>10%) or low distortion *p* value (<0.001) were removed and 13,702 markers were used to construct linkage map for subsequent QTL mapping. Finally, 70 linkage groups consisting of 12,533 SNPs were established on 21 chromosomes (Supplementary Table S1). The entire linkage map spanned 8181.2 cM in total length, with an average distance of 0.7 cM per marker.

### 2.3. QTL Analyses of Three Grain Related-Traits

**Figure 2:**
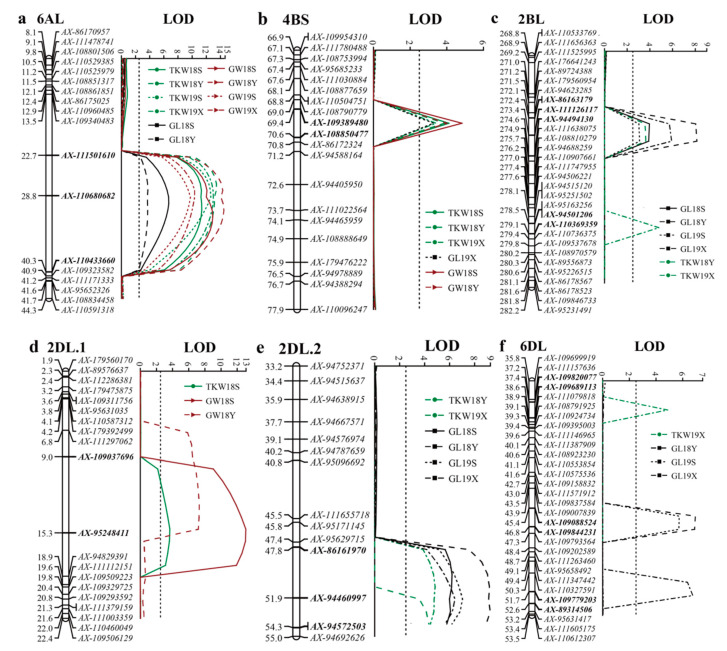
Linkage map and LOD profile of co-located QTLs for thousand kernel weight (TKW), grain length (GL) and grain width (GW) on chromosome 6AL (**a**), 4BS (**b**), 2BL (**c**), 2DL.1 (**d**), 2DL.2 (**e**) and 6DL (**f**), respectively, in the ‘HN5290’ × ‘06Dn23’ recombinant inbred lines (RILs) population; the flanking markers for each QTL are in bold.

A total of 39 additive effect QTLs were identified for TKW, GL and GW in the F_6_ HN5290/06Dn23 population with a 50 K SNP array by using IciMapping software. These QTLs were distributed on almost all 21 chromosomes with exceptions of chromosomes 1D, 3A, 7A and 7D. There were 12 QTLs corresponding to TKW, while 19 QTLs were for GL and 8 QTLs for GW (Supplementary Table S2). Among these loci, 17 loci showed stability in multiple environments and were considered as stable QTLs in below (Table 4 and Figure 2).

### 2.4. Co-Located Loci for All Three Traits

In this study, two co-located loci for the three yield related-traits were identified on chromosome 6AL and 4BS, respectively. These loci were derived from the large grain parent 06Dn23 (Table 4).

QTL *QTkw.haaf-6AL*/*QGl.haaf-6AL*/*QGw.haaf-6AL* was mapped on the long arm of chromosome 6A. It was the most stable locus with the largest phenotypic effect across all of four environments. This co-located locus explained 15.4–24.8% for TKW, 4.1–8.8% for GL and 15.7–24.4% for GW (Table 4). It was flanked by *AX-111501610* and *AX-110680682* markers and located on the physical position at 78.1 Mb-545.7 Mb based on the Chinese Spring wheat reference genome.

The second co-located locus was *QTkw.haaf-4BS*/*Gl.haaf-4BS*/*QGw.haaf-4BS*. It was mapped on wheat chromosome 4BS and flanked by *AX-109389480* and *AX-108850477* markers. This co-located locus explained 8.4–10.2%, 8.8% and 9.3–11.2% phonotypical variation for TKW, GL and GW, respectively (Table 4). It was stably detected in three and two environments for both TKW and GW, but the effect on GL was only detected in one environment. The physical position of this co-located locus was placed at 172.4–345.0 Mb based on the Chinese Spring reference genome (Table 4).

### 2.5. Co-Located Loci for Two Yield Related-Traits

There were 4 loci that were mapped for two yield related-traits among the three and they were identified on the chromosomes 2BL, 2DL (2DL.1 and 2DL.2) and 6DL, respectively. We mapped the effect on TKW at all the four loci, while GL effect was only identified on 2BL, 2DL (2DL.2) and 6DL and GW effect was on 2DL (2DL.1) chromosomes. These loci were also derived from the large grain parent 06dn23 (Table 4). The *QTkw.haaf-2BL*/*QGl.haaf-2BL* was flanked by *AX-86163179* and *AX-110369359* SNP markers and it explained phenotypic variation of 8.5–13.5% for TWK and 9.7–17.8% for GL across all of the four environments (Table 4).

We mapped two co-located loci on the 2DL chromosome, viz. *QTkw.haaf-2DL.1*/*QGw.haaf-2DL.1* and *QTkw.haaf-2DL.2*/*QGl.haaf-2DL.2*. They were located in the interval of *AX-86161970* and *AX-95248411* SNP markers and with a physical distance of 51.6 Mb based on the Chinese Spring reference genome. *QTkw.haaf-2DL.1*/*QGw.haaf-2DL.1* explained 4.6% phenotypic variation for TKW and 10.4–13.8% for GW, and it was present one and two environments, respectively. However, *QTkw.haaf-2DL.2*/*QGl.haaf-2DL.2* explained 9.8–10.7% and 13.6–19.8% phenotypic variation for TKW and GL, respectively. In addition, this co-located locus might be a new one based on previous research and it was stable over 2–4 environments.

*QTkw.haaf-6DL*/*QGl.haaf-6DL* was the last co-located locus in our present study. It was flanked by *AX-109088524* and *AX-89314506* SNP markers and explained 5.3% for TKW and 5.8–7.3% for GL. It was placed at the 384.0–407.5 Mb physical position based on the Chinese Spring reference genome.

### 2.6. The QTL Only Mapped for One Trait Effect

We also mapped one stable locus for TKW, four loci for GL and one locus for GW in the present study and they were distributed on wheat chromosomes 1BS, 2AS, 2AL, 2DS and 5AL, respectively. All of these loci were contributed to by the big grain parent 06dn23 with the exceptions of *QGl.haaf-1BS.1*, *QGl.haaf-2DS* and *QTkw.haaf-5AL* from HN5290.

For the TKW, there was a stable QTL, *QTkw.haaf-5AL*, which was present over three environments. It was flanked by *AX-86165895* and *AX-110976589* SNP markers and explained 5.9–10.7% for TKW.

According to GL, three QTL *QGl.haaf-2AL*, *QGl.haaf-2BL* and *QGl.haaf-2DL.2* were observed on the wheat homoeologous group 2 under all tested environments in the present study and their physical position was co-linear, indicating that there might present three homologous genes controlling GL on group 2 in 06Dn23. They explained 1.8–19.8% phenotype variation of GL under different tested environments. Interestingly, two GL-related loci were located on chromosome 1BS, *QGl.haaf-1BS.1* and *QGl.haaf-1BS.2*, derived from HN5290 and 06Dn23, respectively. The genetic distance between the two QTLs was 4 cM and explained 4.3–6.8% and 6.9–11.3% of phenotypic variance, respectively (Table 4 and Figure 2). Besides, *QGl.haaf-2DS* was detected in over three environments and had minor effects on increasing GL, which can contribute to relatively low phenotypic variance ranging from 3.8 to 6.4% (Table 4).

Only one stable QTL associated with GW was discovered on the chromosome arm 2AS and it was detected in all four environments. It explained phenotypic variance ranging from 8.9 to 14.3%.

### 2.7. Factor ANOVA Analysis between Two Co-Located Loci for Three Traits

In this study, only *QTkw.haaf-4BS*/*Gl.haaf-4BS*/*QGw.haaf-4BS* and *QTkw.haaf-6AL*/*QGl.haaf-6AL*/*QGw.haaf-6AL* were shown to have a co-located effect on three traits. We did factor ANOVA analysis for the gene interaction between these two loci and found that the effect of the single locus was significant (*p* < 0.0001). However, the variation contributed to by the interaction between two loci was 0 (Table 5), and we did not find a significant interaction between them that might due to the additive effect between them. In addition, *QTkw.haaf-4BS* and *QTkw.haaf-6AL* contributed more variation on TKW than the other two traits, which explained 8.4–10.2% and 15.4–24.8%, respectively (Table 4).

## 3. Discussion

### 3.1. Phenotypic Variation Caused by Environments

With global warming and climate change, high temperature poses a great threat to further improve the yield potential for wheat [50,51,52]. Heat stress can reduce grain weight during the grain filling period [12,53,54], resulting in a significant difference for TKW, GL and GW between 2018 and 2019 in the present work that might due to the late planting resulting in the high temperature at the middle and late stage of grain filling in 2018 (Table 1; Figure 1; Supplementary Table S3). Interesting, more QTLs for GL and more stability across different environments were detected than in those of TKW and GW in the present study, indicating that GL was less influenced by environment conditions, as this is determined in the early stage of grain development [8]. In addition, according to Pearson correlation analysis (Table 3), the correlation between TKW and GW is the highest, indicating that GW can reflect grain weight better than GL. Therefore, TKW and GW, with higher coefficient of variation, were more susceptible to the environment in this work. Furthermore, *QGw.haaf-2DL.1* and *QGw.haaf-4BS* were only identified in 2018, which might contribute to the heat tolerance for wheat yield improvement [10].

### 3.2. Comparison of Stable QTL for Grain-Related Traits with Previous Studies

As it is known, grain weight and size are essential components determining wheat yield. Many QTL or homology-based cloned genes for grain-related traits (TKW, GL and GW) have been discovered on all 21 chromosomes [44,55,56]. Based on the genetic analysis of the 194 RIL population of HN5290/06Dn23, 17 stable QTLs were found in chromosome arms 1BS, 2AS, 2AL, 2BL, 2DS, 2DL, 4BS, 5AL, 6AL and 6DL (Supplementary Table S2, Table 4). To identify the relationship of these loci with the previously reported QTLs, the rough physical intervals of QTL regions were obtained by aligning the sequences of markers of the corresponding QTL with the Chinese Spring wheat reference genome [45].

TKW is one of the most direct indicators for wheat yield. Our study showed five stable QTLs for TKW (Table 4 and Figure 2). As expected, most QTLs associated with TKW coincided with GL and/or GW with an exception of the locus on chromosome 5AL, which was consistent with the common agreement that TKW was the core of other grain traits. According to the location of the latest marker *AX-110680682*, the physical location was at ~413 Mb on chromosome 6A, which was located in the same position as the previously reported QTL region (*QTKW-6A-AN* and *QTKW.caas-6AL*) of TKW [23,37,44,57,58]. Likewise, *TaTPP-6AL1* was 13–48 Mb away from these loci and might be the causal gene for this QTL region [44]. However, the effect of this locus on GW and GL has not been reported yet.

The effect of *QTkw.haaf-4BS* on TKW, GL and GW was smaller than the 6A locus. It was located in the physical region at the interval of 172–195 Mb, as this QTL affected TKW by controlling the grain size. There were two major genes for wheat yield, *Rht-B1* and *TB-B1*, which have been reported on the chromosome 4BS [24,59,60]. However, they could be different from *QTkw.haaf-4BS/QGl.haaf-4BS/QGw.haaf-4BS,* at a physical distance of 140 Mb away based on the Chinese Spring reference genome [45]. Chen et al. delimited a QTL for TKW, GL and GW at the 245–432 Mb position of 4BS chromosome [20], which was similar to the *QTkw.haaf-4BS/QGl.haaf-4BS/QGw.haaf-4BS* in present study. Therefore, we speculated that the two QTLs might be the same locus. 

*QTkw.haaf-5AL*, the unique locus related to TKW with its additive from Henong5290, was identified across three environments in the present study and it was almost located in the same position as *QTkw-5A2* and *QTkw.caas-5AL,* as reported by Liu et al. [28,61], respectively. *TaGL3-5A*, a gene controlling grain length in wheat [62], was also located on chromosome 5AL, and was at least 35 Mb away from the closest marker of *QTkw.haaf-5AL*.

*QTkw.haaf-2DL.2* was a novel and stable locus and its effect on TKW and collocation with *QGl.haaf-2DL.2* related to grain length. Xie et al. [8] reported that grain length was determined in the early stage of grain development, while grain width was associated with grain filling in the middle and late stage. In present study, *QTkw.haaf-2DL.2*/ *QGl.haaf-2DL.2* was not associated with grain width. So, this QTL might only be expressed in the early stage of grain development. It was reported that some QTLs related to kernel number per spike and spike number per unit area were located in this region, which were *QSnpp-2D.2*, *QKnps-2D.2*, *QKN-2D-AN*, *QTSS.sicua-2D.2* and *KNS-IWB74*, respectively [44]. These loci are probably controlled by the same gene or genes and further fine mapping is needed.

Grain size was an important part to improving grain yield, which provided space for grain filling [63]. QTL mapping revealed that 12 QTLs associated with grain size were dispersed on chromosomes 1B, 2A, 2B, 2D, 4B, 6A and 6D (Table 4) in present study. The previously reported QTLs or genes were at the similar positions to them. For instance, three stable loci for grain size identified across all environments were separately located on the chromosomes of homologous group 2. Among them, *QGl.haaf-2AL* corresponds to the locus detected in a genome-wide study by Li et al. [29], and within the interval of a cloned gene, *TaFlo2-A1* for TKW [64]. Su et al. [34] and Chen et al. [20] mapped the QTLs of GL and TKW, respectively, which were located in the same interval of *QGl.haaf-2BL*. *QGw.haaf-2DL*, a stable major locus, was located the same position as *qKW-2D.1* reported by Su et al. [34]. In addition, rice gene *Flo2* played a crucial role in regulating grain size [65]. To dissect the relationship between *Flo2* and the latter two loci (*QGl.haaf-2BL* and *QGw.haaf-2DL*), we queried *Flo2* on the online website EnsemblPlants (http://plants.ensembl.org/index.html, (accessed on 1 October 2020)) and obtained the physical positions of orthologs of *Flo2* in wheat (Supplementary Table S4). Noteworthily, the homologous genes *TaFlo2-B1* and *TaFlo2-D1* were also close to *QGl.haaf-2BL* and *QGw.haaf-2DL*, respectively. Furthermore, the expression of three orthologs of *Flo2* was dynamic during the early spike and grain development of bread wheat [66,67,68]. Hence, we speculated that the orthologs of rice *Flo2* controlling grain size are most likely to be the candidate genes for GL- or GW-corresponding QTLs on the chromosomes of wheat homologous group 2 based on the transcriptomic analysis (WheatOmics, http://202.194.139.32/expression/index.html, (accessed on 1 October 2020)). It was reported that *WFZP-2D* has significant effects on spike morphogenesis and yield components [44,69], which was located in the confidence interval of *QGl.haaf-2DS*.

## 4. Materials and Methods

### 4.1. Plant Materials

One hundred and ninety-four F_6_ recombinant inbred lines (RILs) derived from a cross of HN5290 and 06Dn23 were used to do genetic analysis. HN5290, a half-winter wheat cultivar derived from a cross of Shaan 160 × Laizhou 95021, showed an excellent resistance to lodging and fungal disease (yellow rust and powdery mildew). While 06Dn23 showed a higher kernel weight, larger grain size and taller plant height than HN5290. The RIL population was constructed by using single seed descent.

### 4.2. Field Trials and Phenotype Evaluation

The RILs and parents were grown over two cropping seasons at three locations, viz. GaoYi (2018Y), Shijiazhuang (2018S and 2019S) and Xinxiang (2019X). Field trials were carried out around the 10th of October during the cropping season of 2018–2019, while planting was postponed on the 30th of October during the cropping season of 2017–2018 due to the continuous rainfall around the middle of October at Shijiazhuang city. The trials were carried out in a randomized complete block design with two replicates over all environments. Each plot consisted of two 1-m rows with 30 cm spacing.

We harvested the single row of each RIL around the middle of June and mixed threshing. The harvested seeds were dried at room temperature and we measured the TKW, GL and GW every year when the moisture content of the seeds was stabilized around 11–12%. The grain size related-traits were measured using an automatic seed analyzer (SC-G, Wanshen Detection Technology Co., Ltd., Hangzhou, China). GL and GW were calculated by image analysis software after scanning around 500 full-blown grains evenly dispersed on a seed plate, whereas TKW was recorded by weighing above grains using an electronic balance. Finally, the mean of the two replicates in each environment was used for subsequent QTL analysis.

### 4.3. DNA Extraction and Genotyping

Genomic DNA of parents and each RIL was isolated from their leaf using the modified cetyltrimethyl ammonium bromide (CTAB) method [70], and the quality of DNA was appraised using 1% agarose gel electrophoresis. Wheat 50 K SNP array contained over 50,000 SNPs evenly distributed on 21 chromosomes. Some of them were functional markers of cloned genes or closely linked markers to the major loci responsible for agronomic traits and disease resistance [29]. It was designed for breeding selection and developed by the Institute of Crop Science, Chinese Academy of Agricultural Sciences (CAAS). In the present study, the 50 K SNP array was used to genotype the two parents as well as the RIL population. The polymorphic SNPs between parents were used to construct the linkage maps.

### 4.4. Genetic Linkage Maps Construction and QTL Analysis

We selected the polymorphic markers between two parents from the raw SNP data and used BIN functionality of QTL IciMapping 4.2 software (Chinese Academy of Agricultural Sciences, Beijing, China) [71] in order to discard the markers with high missing rate (>10%) and low distortion *p* value (<0.001). Linkage maps were constructed by the same software with the nnTwoOpt method to determine the order of anchors. Based on the anchor results of the marker’s sequence on wheat genome, chromosome location of the remaining linkage groups was identified.

QTL analysis was carried out following the BIP functionality of QTL IciMapping 4.2 software by the inclusive composite interval mapping with additive effect (ICIM-ADD) method. The mapping parameters were set as step = 1.0 cM, PIN = 0.001, and LOD threshold was 3.0 by manual input. QTLs were named according to McCouch’s method [72].

### 4.5. Statistical Analysis

Fundamental data analyses, including descriptive statistics, phenotypic correlation analysis, analysis of variance (ANOVA), normality test and *t*-test, were conducted using SPSS 20.0 software (SPSS, Chicago, IL, USA). The formula *H*^2^ = *σ*^2^*_g_*/(*σ*^2^*_g_*+*σ*^2^*_ge_*/*m*+*σ*^2^*_gy_*/*n*+*σ*^2^/*nr*) was used to estimate the broad-sense heritability. *H*^2^ represents each trait with the Ime4 package in R 3.5 software. *σ*^2^*_g_*, *σ*^2^*_ge_* and *σ*^2^*_gy_* represent the variance of genotype, genotype × environment interaction and genotype × year, respectively, whereas *m*, *n* and *r* represent the number of environments (locations), cropping seasons and replicates, *σ^2^* is the residual error. The linkage map and LOD profile of QTLs were drawn using MapChart 2.32 software. SAS version 9.3 was used for ANOVA and correlation analysis with TKW data in each environment.

## 5. Conclusions

HN5290 was a new wheat variety bred by the Wheat Breeding Laboratory of Hebei Agricultural University. It was highly resistant to stripe rust and moderately resistant to leaf rust and was a high-yield and high-quality wheat variety. 06dn23 was a wheat line which has not been released in Hebei Province. It had large grain, high TKW and higher plant height than HN5290. The two parents had good agronomic characters and had big potential to be used in wheat breeding. Maintaining high and stable yield of wheat had always been the goal of breeders. The grain length of wheat played an important role in increasing TKW. The new locus *QGl.haaf-2DL.2* in this study could significantly increase the grain length of wheat, and indirectly increase the yield of wheat. The tightly linked marker of *QTkw.haaf-2DL.2*/*QGl.haaf-2DL.2* could be used in wheat molecular breeding.

## Figures and Tables

**Table 1:** Phenotypic performance for thousand kernel weight (TKW), grain length (GL) and grain width (GW) of the F_6_ recombinant inbred lines (RILs) and their parents (HN5290 and 06Dn23).

Trait ^a^	Environment ^b^	HN5290	06Dn23	Signi. ^d^	194 RILs Population		
		Mean ± SD ^c^	Mean ± SD		Mean ± SD	Range	CV ^e^ (%)
TKW (g)	18Y	25.91 ± 1.89	46.29 ± 7.25	***	37.62 ± 5.45	20.99–49.70	14.49
	18S	30.00 ± 3.96	51.46 ± 4.67	***	36.96 ± 5.85	17.59–49.71	15.83
	19X	45.78 ±1.22	70.79 ±2.12	***	57.81 ± 4.45	40.28–66.97	7.70
	19S	41.66 ± 2.72	59.44 ± 3.16	***	53.06 ± 5.61	31.96–64.89	10.57
GL (mm)	18Y	6.08 ± 0.06	7.52 ± 0.18	***	7.10 ± 0.29	6.34–7.84	4.02
	18S	6.30 ± 0.13	7.73 ± 0.20	***	6.85 ± 0.31	5.92–7.53	4.51
	19X	6.67 ± 0.07	8.33 ± 0.08	***	7.59 ± 0.30	6.80–8.42	3.90
	19S	6.64 ± 0.12	8.11 ± 0.14	***	7.60 ± 0.30	6.60–8.45	3.97
GW (mm)	18Y	2.79 ± 0.05	3.37 ± 0.15	***	3.11 ± 0.16	2.56–3.53	5.30
	18S	2.88 ± 0.12	3.44 ± 0.11	***	3.09 ± 0.16	2.57–3.41	5.19
	19X	3.39 ± 0.04	3.89 ± 0.06	***	3.65 ± 0.11	3.23–3.93	3.03
	19S	3.23 ± 0.10	3.63 ± 0.07	***	3.50 ± 0.15	2.89–3.82	4.17

^a^ TKW, one-thousand kernel weight; GL, grain length; GW, grain width. ^b^ 18 and 19 represent 2017–2018 and 2018–2019 cropping season, respectively; Y, GaoYi, China; S, Shijiazhuang, China; X, XinXiang, China. ^c^ SD, standard deviation. ^d^ Signi., Significance level of difference between parents. ^e^ CV, coefficient of variation in percent. ***, *p* < 0.001.

**Table 2:** Analysis of variance (ANOVA) and broad sense heritability for thousand kernel weight (TKW), grain length (GL) and grain width (GW) in 194 recombinant inbred lines (RILs) population over 4 environments.

Source of Variation	df	Mean Square		
		TKW	GL	GW
Environments	3	43,584.04 ***	52.32 ***	31.11 ***
Lines	193	179.50 ***	0.62 ***	0.13 ***
Lines * Environments	579	16.44 ***	0.03	0.02
Error	768	6.82	0.01	0.01
Heritability		0.80	0.89	0.75

*** Significant at the 0.001 probability level.

**Table 3:** Correlation coefficients among 12 environmental phenotypic data in an F_6_ recombinant inbred lines (RILs) population derived from a cross of HN5290 × 06Dn23.

Environment	TKW18Y ^a^	TKW18S ^b^	TKW19X ^c^	TKW19S	GL ^d^ 18Y	GL18S	GL19X	GL19S	GW ^e^ 18S	GW18Y	GW19X
TKW18S	0.80 **										
TKW19X	0.71 **	0.69 **									
TKW19S	0.70 **	0.67 **	0.77 **								
GL18Y	0.66 **	0.61 **	0.66 **	0.61 **							
GL18S	0.59 **	0.71 **	0.66 **	0.61 **	0.87 **						
GL19X	0.47 **	0.49 **	0.71 **	0.51 **	0.83 **	0.82 **					
GL19S	0.42 **	0.47 **	0.59 **	0.65 **	0.81 **	0.78 **	0.81 **				
GW18S	0.76 **	0.94 **	0.62 **	0.61 **	0.46 **	0.57 **	0.32 **	0.32 **			
GW18Y	0.92 **	0.71 **	0.62 **	0.60 **	0.44 **	0.39 **	0.25 **	0.21 **	0.78 **		
GW19X	0.57 **	0.54 **	0.82 **	0.62 **	0.33 **	0.35 **	0.28 **	0.25 **	0.62 **	0.65 **	
GW19S	0.62 **	0.57 **	0.63 **	0.88 **	0.38 **	0.40 **	0.22 **	0.34 **	0.61 **	0.64 **	0.69 **

^a^ TKW, one-thousand kernel weight, 18 represents the crop season of 2017–2018; Y, GaoYi, China; ^b^ S, Shijiazhuang, China; ^c^ X, XinXiang, China; 19 represents the crop season of 2018–2019; ^d^ GL, grain length; ^e^ GW, grain width; ** *p* < 0.01.

**Table 4:** Stable QTLs for thousand kernel weight (TKW), grain length (GL) and grain width (GW) in the F_6_ recombinant inbred lines (RILs) population derived from a cross of HN5290 × 06Dn23 over all tested environments by using Inclusive Composite Interval Mapping software.

Traits	QTL	Environment	Position	Left Marker	Right Marker	LOD ^a^	PVE (%) ^b^	Add ^c^
TKW	*QTkw.haaf-2BL*	18Y	274	*AX-111126117*	*AX-94494130*	3.6	8.5	−1.60
		19X	279	*AX-94501206*	*AX-110369359*	4.8	13.5	−1.47
	*QTkw.haaf-2DL.1*	18S	15	*AX-109037696*	*AX-95248411*	3.7	4.6	−1.21
	*QTkw.haaf-2DL.2*	18Y	54	*AX-94460997*	*AX-94572503*	4.3	9.8	−1.73
		19X	51	*AX-86161970*	*AX-94460997*	4.8	10.7	−1.51
	*QTkw.haaf-4BS*	18S	70	*AX-109389480*	*AX-108850477*	4.1	10.2	−1.79
		18Y	70	*AX-109389480*	*AX-108850477*	3.9	9.5	−1.62
		19X	70	*AX-109389480*	*AX-108850477*	3.4	8.4	−1.25
	*QTkw.haaf-5AL*	18S	187	*AX-86165895*	*AX-110976589*	5.1	6.5	1.47
		19S	186	*AX-109966154*	*AX-86165895*	4.9	5.9	1.41
		19X	187	*AX-86165895*	*AX-110976589*	9.4	10.7	1.47
	*QTkw.haaf-6AL*	18S	31	*AX-110680682*	*AX-110433660*	11.4	17.2	−2.40
		18Y	27	*AX-111501610*	*AX-110680682*	13.4	24.8	−2.58
		19S	29	*AX-111501610*	*AX-110680682*	12.6	16.6	−2.39
		19X	29	*AX-111501610*	*AX-110680682*	13.0	15.4	−1.78
	*QTkw.haaf-6DL*	19X	38	*AX-109820077*	*AX-109689113*	4.9	5.3	−1.03
GL	*QGl.haaf-1BS.1*	18S	99	*AX-94535608*	*AX-86174948*	24.9	5.3	0.26
		18Y	99	*AX-94535608*	*AX-86174948*	21.5	4.3	0.22
		19S	99	*AX-94535608*	*AX-86174948*	30.7	6.8	0.29
		19X	99	*AX-94535608*	*AX-86174948*	24.5	5.3	0.25
	*QGl.haaf-1BS.2*	18S	103	*AX-179477422*	*AX-179476084*	35.0	8.5	−0.33
		18Y	103	*AX-179477422*	*AX-179476084*	30.3	6.9	−0.28
		19S	103	*AX-179477422*	*AX-179476084*	42.9	11.3	−0.37
		19X	103	*AX-179477422*	*AX-179476084*	35.5	8.9	−0.32
	*QGl.haaf-2AL*	18S	16	*AX-108902945*	*AX-111489408*	5.3	7.3	−0.08
		18Y	18	*AX-108902945*	*AX-111489408*	5.5	6.3	−0.07
		19S	17	*AX-108902945*	*AX-111489408*	5.3	1.8	−0.07
		19X	16	*AX-108902945*	*AX-111489408*	5.3	5.6	−0.06
	*QGl.haaf-2BL*	18S	273	*AX-86163179*	*AX-111126117*	4.0	11.9	−0.09
		18Y	274	*AX-111126117*	*AX-94494130*	8.1	17.8	−0.12
		19S	274	*AX-111126117*	*AX-94494130*	3.1	9.7	−0.08
		19X	274	*AX-111126117*	*AX-94494130*	5.9	16.5	−0.10
	*QGl.haaf-2DS*	18Y	1	*AX-111112187*	*AX-179558004*	3.8	3.8	0.05
		19S	1	*AX-111112187*	*AX-179558004*	6.4	6.4	0.08
		19X	7	*AX-109634352*	*AX-110507164*	6.2	5.4	0.07
	*QGl.haaf-2DL.2*	18S	50	*AX-86161970*	*AX-94460997*	6.2	13.6	−0.12
		18Y	53	*AX-94460997*	*AX-94572503*	9.2	19.8	−0.13
		19S	52	*AX-94460997*	*AX-94572503*	7.0	15.4	−0.12
		19X	52	*AX-94460997*	*AX-94572503*	6.4	17.6	−0.10
	*QGl.haaf-4BS*	19X	70	*AX-109389480*	*AX-108850477*	3.3	8.8	−0.08
	*QGl.haaf-6AL*	18S	30	*AX-110680682*	*AX-110433660*	6.7	8.8	−0.09
		18Y	28	*AX-111501610*	*AX-110680682*	3.7	4.1	−0.05
	*QGl.haaf-6DL*	18Y	46	*AX-109088524*	*AX-109844231*	6.9	7.3	−0.07
		19S	46	*AX-109088524*	*AX-109844231*	5.7	5.8	−0.08
		19X	52	*AX-109779203*	*AX-89314506*	6.8	5.9	−0.07
GW	*QGw.haaf-2AS*	18S	15	*AX-110478994*	*AX-111530828*	4.7	10.5	−0.05
		18Y	15	*AX-110478994*	*AX-111530828*	3.9	8.9	−0.05
		19S	15	*AX-110478994*	*AX-111530828*	6.5	14.3	−0.06
		19X	15	*AX-110478994*	*AX-111530828*	5.3	11.7	−0.04
	*QGw.haaf-2DL.1*	18S	15	*AX-109037696*	*AX-95248411*	13.1	13.8	−0.06
		18Y	13	*AX-109037696*	*AX-95248411*	7.3	10.4	−0.05
	*QGw.haaf-4BS*	18S	70	*AX-109389480*	*AX-108850477*	4.8	11.2	−0.05
		18Y	70	*AX-109389480*	*AX-108850477*	4.0	9.3	−0.05
	*QGw.haaf-6AL*	18S	32	*AX-110680682*	*AX-110433660*	12.9	15.7	−0.07
		18Y	31	*AX-110680682*	*AX-110433660*	14.5	24.4	−0.08
		19S	29	*AX-111501610*	*AX-110680682*	9.8	19.3	−0.06
		19X	28	*AX-111501610*	*AX-110680682*	10.4	15.9	−0.05

^a^ Logarithm of odds (LOD) score of QTL peak. ^b^ Proportion of phenotypic variance explained by each QTL. ^c^ Additive effect for the QTL; positive values indicate the effect of HN5290 alleles, whereas negative values indicate that the alleles came from 06Dn23.

**Table 5:** Factor ANOVA analysis of two co-located loci for thousand kernel weight (TKW), grain length (GL) and grain width (GW) on the chromosomes 4BS and 6AL.

Source	df	Type III SS ^a^	Mean Square	F Value	*p* > *F* ^b^	Variation (%)
Year	2	54,492.3	27,246.1	1338.1	<0.0001	80.9
*QTkw.haaf-4BS*	3	1079.9	534.0	26.5	<0.0001	3.0
*QTkw.haaf-6AL*	3	1533.6	766.8	37.7	<0.0001	4.4
*QTkw.haaf-4BS*& *QTkw.haaf-6AL*	3	48.6	16.2	0.8	0.4966	0
Year	1	6.1	6.1	80.7	<0.0001	23.6
*QGL.haaf-4BS*	2	2.0	1.0	13.5	<0.0001	9.0
*QGL.haaf-6AL*	2	1.3	0.6	8.4	0.0003	9.7
*QGL.haaf-4BS*& *QGL.haaf-6AL*	2	0.1	0.1	0.2	0.9271	0
Year	1	0.1	0.1	2.1	0.1441	0.4
*QGW.haaf-4BS*	2	0.8	0.4	20.8	<0.0001	17.4
*QGW.haaf-6AL*	2	0.7	0.3	16.7	<0.0001	17.7
*QGW.haaf-4BS*& *QGW.haaf-6AL*	2	0.1	0.1	1.0	0.4233	0

^a^ Type III SS = the third type of sum of squares. ^b^ *p* < 0.0001.

## Data Availability

The data presented in this study are available on request from the corresponding author. The data are not publicly available due to the related research is under going for other traits.

## Supplementary Materials

The following are available online at https://www.mdpi.com/2223-7747/10/4/713/s1, Table S1: Descriptive statistics of 70 linkage groups across 21 wheat chromosomes, Table S2: QTL for thousand kernel weight (TKW), grain length (GL) and grain width (GW) over all tested environments by using Inclusive Composite Interval Mapping software, Table S3: Comparison of TGW, GL and GW between the RILs with ‘HN5290’ genotype and ‘06Dn23’ genotype for two reliable QTL on chromosome 2BL and 6AL in the F_6_ RILs population derived from HN5290×06Dn23, Table S4: Approximate physical positions of three QTL for grain size on homologous chromosome group 2 and three orthologs of rice Flo2 in wheat, Table S5: Highest and lowest temperature of one day during wheat grain filling period in different environments.
